# Spatial pattern evolution and regional interaction mechanisms of sports and recreation facilities in jiangsu province, China: a gis-based spatial econometric analysis

**DOI:** 10.1186/s12942-026-00472-8

**Published:** 2026-05-24

**Authors:** Xin Lyu, Yanling Li, Yuming Tai

**Affiliations:** 1 School of Physical Education and Sports, Soochow University, 50 Donghuan Road, Suzhou, 215021 Jiangsu Province China; 2 College of Physical Education, Chuzhou University, No. 1, Huifeng West Road, Chuzhou, 239000 Anhui Province China

**Keywords:** Sports and recreation facilities, Spatiotemporal dynamics, Spatial spillover effects, GIS, Spatial durbin model

## Abstract

The equitable allocation of public service facilities is a key issue in regional coordinated development; however, the long-term spatiotemporal dynamics of their spatial distribution and interregional interaction mechanisms remain underexplored. Based on point-of-interest (POI) data from 13 prefecture-level cities in Jiangsu Province, this study employs exploratory spatial data analysis (ESDA) methods and the spatial Durbin model to investigate the evolutionary patterns and driving mechanisms of sports and recreation facility allocation from 2014 to 2023. The findings reveal that the distribution of sports and recreation facilities in Jiangsu exhibits a stable core-periphery structure, yet limited diffusion is evident, characterized by the sprawling of core areas, the formation of corridors along the Yangtze River, and the emergence of growth poles in peripheral regions. The spatial pattern is undergoing a balanced reconstruction from linear agglomeration to a “from-line-to-area” configuration. Regarding driving mechanisms, the spatial externalities of different factors exhibit significant differentiation: economic growth generates positive synergistic spillovers, while population density, industrial agglomeration, and road density manifest as negative spillovers through siphon effects, crowding-out effects, and consumption substitution, respectively. This dynamic structure helps explain the underlying mechanisms sustaining the highly stable macro pattern. This study is an attempt to reveal the duality of spatial spillover effects of public service facilities with a progressive analytical paradigm. The empirical evidence from Jiangsu Province may provide useful references for the optimization of facility layout and spatial governance in other regions.

## Introduction

In the context of rapid urbanization, urban infrastructure development has long prioritized “quantity attainment” over the “quality and efficiency” of facility layout [1]. This orientation tends to lead to multiple challenges, including widened regional service gaps, imbalanced spatial resource allocation, and low facility utilization efficiency [2]. As fundamental spatial carriers of public sports activities, the allocation of sports and recreation facilities has received increasing attention. With the implementation of the “Healthy China 2030” strategy, public concern has shifted from “whether there are facilities” to “whether facility layout is reasonable and usage is convenient,” raising higher requirements for the spatial allocation efficiency of sports and recreation facilities. Against this background, the successive issuance of policy documents—including the *Outline for Building a Leading Sports Nation* [3], the *National Fitness Plan (2021–2025)* [4], and the *Work Plan for the Fitness Facilities Enhancement Initiative (2023–2025)* [5]—reflects a policy shift from mere scale expansion toward the integrated consideration of quality improvement and spatial balance. This underscores the growing importance of optimizing facility layout and promoting coordinated regional development.

Existing studies on the spatial allocation of sports and recreation facilities have primarily focused on two dimensions. The first adopts a geographical perspective, employing GIS-based spatial analysis to describe distribution patterns, agglomeration characteristics, and accessibility levels [6–8]. The second draws on public management or economics perspectives, examining the effects of single or limited factors, such as population structure, economic development, and fiscal capacity on facility supply [9–12]. Some studies have further addressed supply-demand matching [13, 14], particularly accessibility issues for vulnerable groups [15–17].While these studies have laid a foundation for understanding static characteristics and local linkages, a critical yet relatively neglected perspective is that in highly interconnected regional systems, facility allocation in any given area is not an isolated decision but rather involves complex spatial interactions with neighboring regions [18–22]. Although some scholars have begun to explore spatial interactions in public services [23–25], most studies remain implicitly grounded in the assumption of “regional independence,” failing to incorporate spatial dependence and spillover effects into their analytical core. This limits the ability to reveal the underlying mechanisms of “synergistic development” or “competitive differentiation” between regions.

**Fig. 1:**
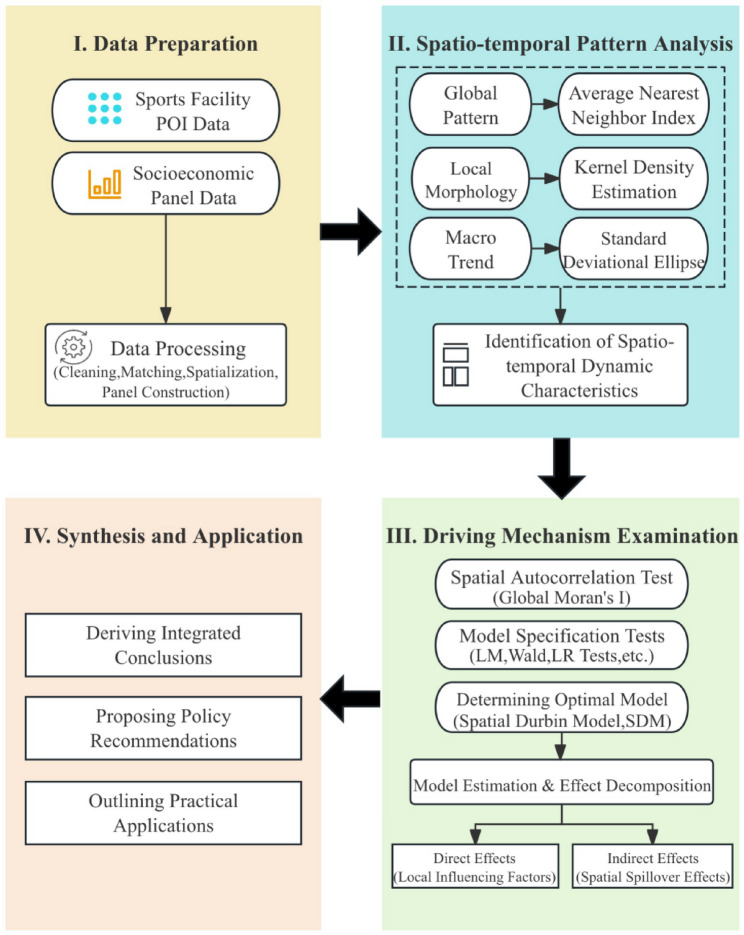
Research methodology and technical flowchart. This diagram outlines the complete analytical workflow, from data preparation through to the synthesis of findings. It delineates the sequential phases of data processing, exploratory spatial analysis, econometric modeling, and the final integration of results to draw theoretical and practical conclusions

As a pioneer in sports and recreation facility development in China, Jiangsu Province achieved a per capita facility area of 4.46 square meters by the end of 2024, ranking first nationwide [26]. In terms of economic development, the per capita GDP of Southern Jiangsu was 201,400 yuan in 2024, compared to 108,100 yuan in Northern Jiangsu [27]. Given that the allocation of public service facilities is highly dependent on the local economic base, these economic disparities profoundly influence the spatial differentiation of sports and recreation facilities. The coexistence of “national leadership” and “significant internal gradients” makes Jiangsu an ideal case for investigating the spatial allocation of public service facilities and their regional interaction mechanisms. Accordingly, this study focuses on addressing three specific questions: (1) What evolutionary patterns characterize the spatial distribution of sports and recreation facilities in Jiangsu Province from 2014 to 2023? (2) What socioeconomic factors drive these spatial patterns, and what are the nature and magnitude of their local and spatial spillover effects? (3) What policy implications do these findings have for promoting coordinated allocation of regional sports resources? To address these questions, this study follows a “pattern identification – mechanism testing – effect decomposition” framework (Fig. 1). Exploratory spatial data analysis is first employed to characterize pattern evolution; the spatial Durbin model is then applied to examine the local and spillover effects of driving factors; finally, empirical findings are synthesized to distill theoretical insights and practical implications.

## Theoretical foundation

### Local dynamics and spatial spillover mechanisms

As quasi-public goods [28, 29], sports and recreation facilities are not randomly distributed but are deeply embedded in the regional socioeconomic environment, shaped by multiple driving forces. To systematically disentangle their underlying mechanisms, this study develops a theoretical foundation from three interrelated dimensions.

1. **Demand serves as the primary driving force and structural impetus for spatial agglomeration.** Public goods theory suggests that the provision of public services exhibits significant economies of scale: population agglomeration both generates demand and reduces per-unit service costs, thereby incentivizing facility concentration in population centers [30]. This directs our attention to population density as a core indicator of basic demand scale. Demographic transition theory further reveals that urbanization entails fundamental lifestyle changes, giving rise to “derived demand” for high-quality sports services [31], positioning urbanization rate as a key variable capturing demand structure upgrading. Moreover, according to consumer behavior theory and the home market effect in new economic geography, when demand for specific services cannot be adequately met locally, residents may cross administrative boundaries to consume in neighboring areas, generating “demand spillovers” [32, 33]. This implies that neighboring regions’ population sizes and demand structures may also generate industrial linkages with the local area through consumption flows.
2. **Supply functions as capacity constraints and behavioral choices in resource allocation.** As higher-order needs, sports consumption relies heavily on a solid economic foundation. A region’s economic development level (GDP) constitutes the common cornerstone of both residents’ consumption capacity and government fiscal capacity. Fiscal decentralization theory emphasizes that local governments, as primary suppliers of local public goods, directly reflect their supply preferences, fiscal efforts, and policy priorities under resource constraints through the size and structure of their general public budget expenditures. At the regional level, local governments engage in strategic interactions in public service provision. When neighboring areas successfully construct large sports facilities, their experiences may be borrowed through policy learning or demonstration effects. Simultaneously, under performance evaluation pressure, local governments may benchmark their public service levels against neighboring areas through yardstick competition [34, 35].
3. **Environment acts as the spatial carrier and ecological foundation for facility location.** Cost and accessibility theory emphasizes that infrastructure networks directly determine construction costs and residents’ spatial-temporal resistance, serving as the hardware foundation shaping the economic logic of facility location [36]. Agglomeration economy theory reveals the enabling role of industrial ecology: spatial concentration of employees in related sectors promotes knowledge spillovers and forms specialized markets, generating positive externalities for facility supply [37].Infrastructure and industrial agglomeration exhibit cross-regional network externalities [38]. Improvements in road density in neighboring areas may enhance overall regional accessibility, indirectly affecting local facility location efficiency and service coverage [39]. Similarly, agglomeration effects in sports-related industries may transcend administrative boundaries, with knowledge spillovers and labor pooling radiating to neighboring areas.

### Theoretical framework construction

**Fig. 2:**
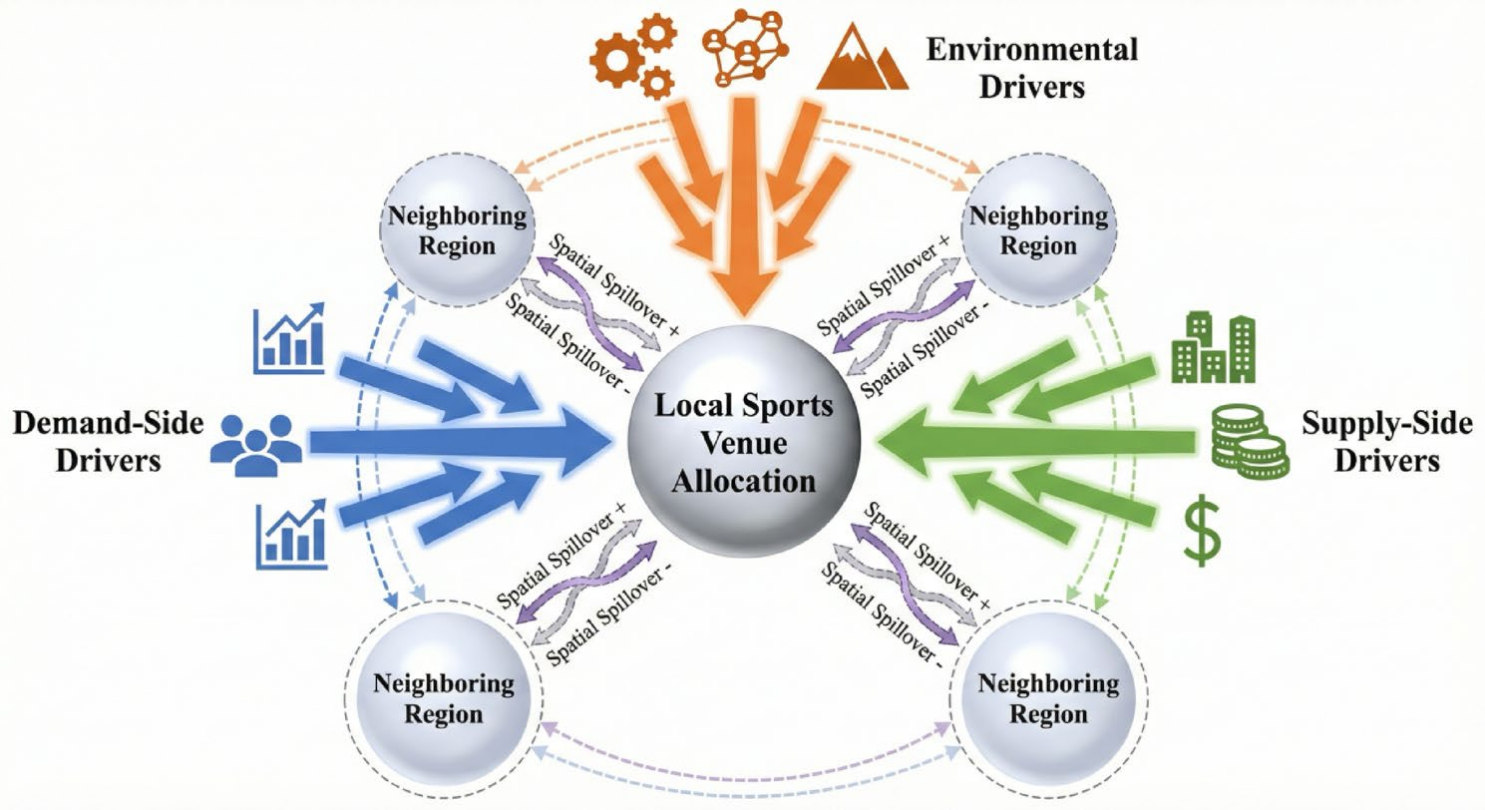
Analytical framework of three-dimensional drivers and spatial spillover effects for sport and recreation facility spatial allocation. The demand side, supply side, and environmental support side jointly shape the allocation of sports and recreation facilities. Potential interaction effects may exist among these three dimensions, which can influence neighboring regions through spatial spillover channels

In summary, this study constructs a three-dimensional theoretical framework for sports and recreation facility allocation, centered on the core elements of “demand side, supply side, and environmental support side.” The framework posits that the configuration of local sports and recreation facilities is the outcome of the interaction and equilibrium among local demand gravity, supply forces, and environmental enablers. In highly interconnected regional economic systems, the influence of these factors inevitably generates a degree of spatial interaction—local facility allocation is shaped not only by its own conditions but also by the corresponding characteristics of neighboring regions (Fig. 2). This cross-regional influence constitutes spatial spillover effects, whose specific directions (synergistic promotion or competitive inhibition) and magnitudes form the core mechanisms this study seeks to uncover.

## Study area, data, and methods

### Study area overview

**Fig. 3:**
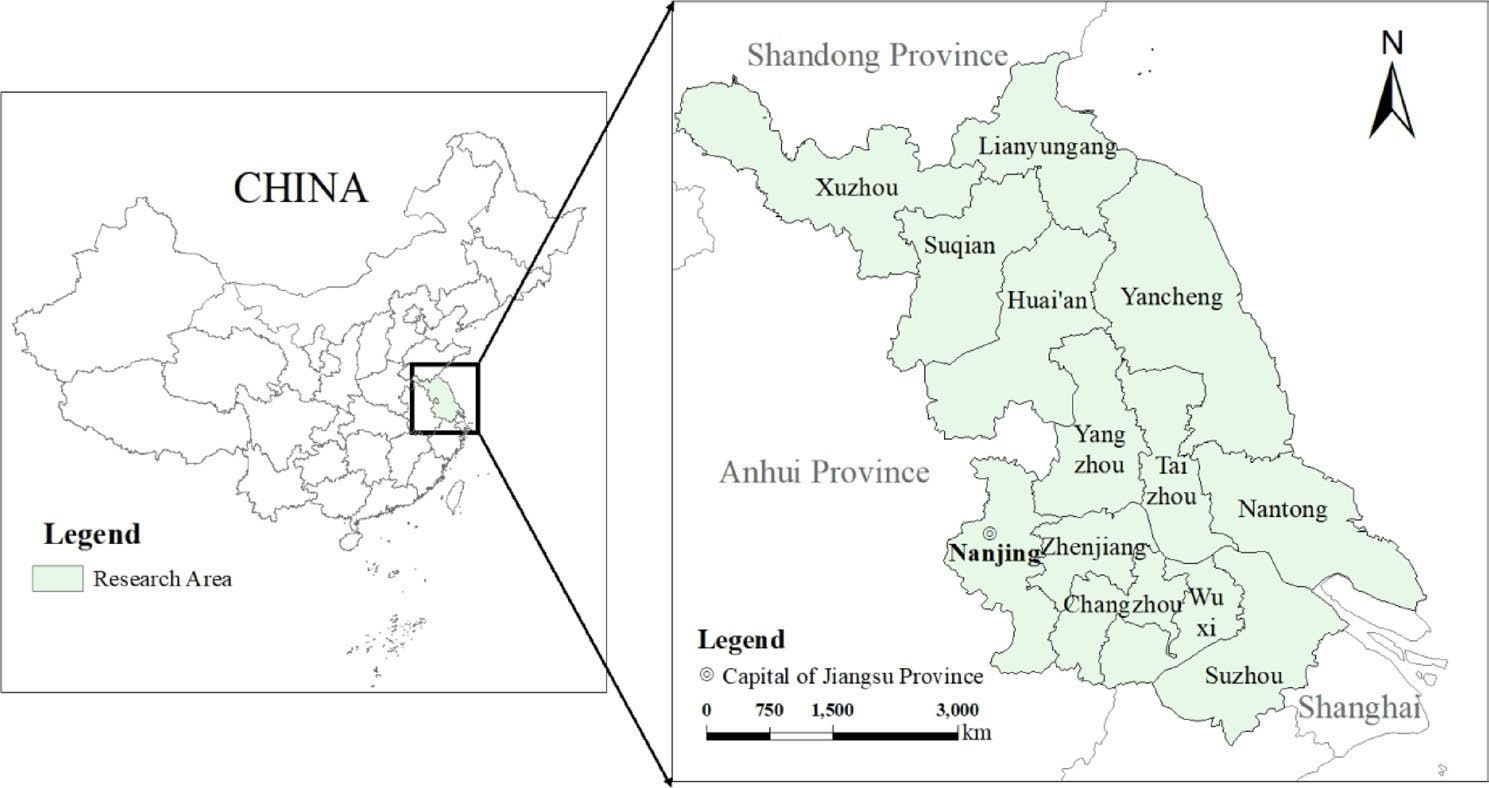
Location of the study area (Jiangsu Province, China)

The study area, Jiangsu Province, comprises 13 prefecture-level cities (Fig. 3), which are conventionally divided into three major regions: Southern Jiangsu (Nanjing, Suzhou, Wuxi, Changzhou, Zhenjiang), Central Jiangsu (Nantong, Yangzhou, Taizhou), and Northern Jiangsu (Xuzhou, Lianyungang, Huai’an, Yancheng, Suqian). These regions exhibit distinct gradients in economic development stages while maintaining close connections under the integration of the Yangtze River Delta Region, resulting in significant spatial interactions. Therefore, selecting this case study helps to more effectively identify the local factors and spatial spillover effects influencing sports and recreation facility allocation while controlling for the broader regional context.

### Data sources and processing

#### Point-of-Interest (POI) data

The dependent variable was constructed using annual point-of-interest (POI) data for sports and recreation facilities in Jiangsu Province from 2014 to 2023.These data were systematically collected through the application programming interfaces (APIs) of Amap and Baidu Maps, using search keywords including “stadiums,” “fitness centers,” “gyms,” “sports parks,” “basketball courts,” “football fields,” “badminton halls,” and “swimming pools.” To ensure data quality, the raw data underwent coordinate correction, deduplication, and manual verification. The cleaned point data were then spatially aggregated to the 13 prefecture-level cities by year, yielding the total annual count of sports and recreation facility POIs for each city.

#### Socioeconomic panel data

The explanatory variables were constructed using prefecture-level panel data from the Jiangsu Statistical Yearbook and municipal statistical yearbooks (2015–2024 editions). Given the publication lag (i.e., the year T volume publishes data for year T-1), data corresponding to 2014–2023 were extracted, including annual end-of-year resident population, gross regional product, general public budget expenditure, urbanization rate, road mileage, and employment in culture, sports, and entertainment. Linear interpolation was employed to fill missing data for individual years and counties/districts.

#### Basic geographic data

Administrative boundary vector data for Jiangsu Province and its 13 prefecture-level cities were obtained from the National Geomatics Center of China for spatial georeferencing and unit delineation.

### Research methods

#### Exploratory spatial data analysis methods

1. Global spatial pattern identification: average nearest neighbor index

The Average Nearest Neighbor (ANN) index determines the overall spatial distribution pattern (clustered, random, or dispersed) of point features by comparing the average observed distance between each facility point and its nearest neighbor with the expected average distance under a completely random spatial distribution. Its calculation formula is:

1 $$\:\mathrm{ANN=}\frac{{\bar{D}}_o}{{\bar{D}}_E}$$

where ${{\bar{D}}_o}$ is the average observed distance between each sports and recreation facility point and its nearest neighbor, and ${{\bar{D}}_E}$ is the expected average distance under a random distribution pattern ($\:{{\bar{D}}_E}\mathrm{=0.5/}\sqrt{\mathrm{n/A}}$, where *n* is the number of points and *A* is the study area). *ANN* < 1 indicates a clustered distribution, *ANN* ≈ 1 suggests a random distribution, and *ANN* > 1 implies a dispersed distribution. Statistical significance is assessed using the Z-score test. The ANN index was calculated annually to reveal its dynamic changes over time.

2. Local spatial agglomeration morphology: kernel density estimation

Kernel Density Estimation (KDE) is used to visualize the local density distribution and agglomeration hotspots of sports and recreation facilities in space. Its formula is:

2 $$\:\widehat{\mathrm{f}}\left(\mathrm{x}\right)\mathrm{=}\frac{\mathrm{1}}{\mathrm{nh}}\sum\:_{\mathrm{i}\mathrm{=1}}^{\mathrm{n}}\mathrm{K}\left(\frac{\mathrm{d}\left(\mathrm{x}\mathrm{,}{\mathrm{x}}_{\mathrm{i}}\right)}{\mathrm{h}}\right)$$

where $\:\widehat{\mathrm{f}}\left(\mathrm{x}\right)\text{}$ is the estimated density at location *x*, *n* is the number of sports and recreation facility points, *K* is the kernel function (a quadratic kernel function is used in this study), $\:\mathrm{d}\left(\mathrm{x}\mathrm{,}{\mathrm{x}}_{\mathrm{i}}\right)$is the Euclidean distance between point *x* and facility point $\:{\mathrm{x}}_{\mathrm{i}}$, and the bandwidth *h* is optimized using Silverman’s rule-of-thumb. By comparing multi-temporal KDE maps, the spatiotemporal evolution of hotspot areas is identified.

3. Macro-level distributional trends: standard deviational ellipse

The Standard Deviational Ellipse (SDE) geometrically summarizes the spatial distribution characteristics of point sets, including central tendency, dominant direction, degree of dispersion, and spatial extent. The core parameters of the ellipse are described in Table 1.

**Table 1:** Description of core parameters of the standard deviational ellipse

Core parameter	Symbol	Spatial meaning
Center	$\:\left(\overline{X},\,\,\overline{Y}\right)$	Represents the mean center of the spatial point distribution, reflecting the “central” tendency of resource allocation.
Rotation angle	$\:\theta$	Indicates the dominant direction of the distribution, measured clockwise from north (0°).
Major axis standard deviation	$\sigma_x$	Measures the dispersion of points along the dominant direction; larger values indicate a wider extent.
Minor axis standard deviation	$\sigma_y$	Measures the dispersion of points along the secondary direction; a larger difference between $\sigma_x \,\,and \,\,\sigma_y$ indicates a stronger directional trend.
Ellipse area	*S*	Represents the overall coverage extent of the distribution, determined jointly by$\sigma_x \,\,and \,\,\sigma_y$.

#### Spatial econometric models

1. Variable selection and indicator system construction

This study uses the annual count of sports and recreation facility POIs in each city as the dependent variable, applying a natural logarithm transformation (denoted as *lny*) to mitigate heteroscedasticity. The specific composition and measurement of the explanatory variables for each dimension are presented in Table 2. To eliminate dimensional differences, all continuous variables were standardized (Z-score) prior to estimation.

2. Basic specification of spatial econometric models

To more accurately capture the complex economic-geographic interactions among prefecture-level cities[40–41], this study constructs an economic-geographic weight matrix W. This matrix incorporates both geographical distance decay effects and the similarity in economic development levels. Its element "w" _"ij" is defined as follows:

**Table 2:** Variable indicator system and operational definitions

Theoretical dimension	Core mechanism	Variable	Symbol	Operational definition
**Demand-side drivers**	Scale demand	Population density	*lnPopDen*	Natural logarithm of (year-end resident population/administrative area)
	Structural upgrading	Urbanization rate	*UrbanRate*	Urban population/Total population (%)
**Supply-side drivers**	Economic support	Gross regional product	*lnGDP*	Natural logarithm of Gross Regional Product (GDP)
	Fiscal input	General public budget expenditure	*lnFiscalExp*	Natural logarithm of general public budget expenditure
**Environmental-support drivers**	Facility carrying capacity	Road network density	*RoadDen*	Total road mileage/Administrative area
	Industrial synergy	Employment in culture, sports, and entertainment	*lnIndEmp*	Natural logarithm of the number of employees in culture, sports, and entertainment

3 $$\:{\mathrm{w}}_{\mathrm{ij}}\mathrm{=}\frac{\left(\mathrm{1/}{\mathrm{d}}_{\mathrm{ij}}\right)\cdot\left(\mathrm{1/}\left|{\overline{\mathrm{PGDP}}}_{\mathrm{i}}-{\overline{\mathrm{PGDP}}}_{\mathrm{j}}\right|\right)}{\sum\:_{\mathrm{j}\neq\mathrm{i}}\left(\mathrm{1/}{\mathrm{d}}_{\mathrm{ij}}\right)\cdot\left(\mathrm{1/}\left|{\overline{\mathrm{PGDP}}}_{\mathrm{i}}-{\overline{\mathrm{PGDP}}}_{\mathrm{j}}\right|\right)}\text, \,\,\mathrm{i}\neq\mathrm{j}$$

where $\:{\mathrm{d}}_{\mathrm{ij}}\:$is the Euclidean geographical distance calculated based on the coordinates of prefecture-level city government seats; $\:{\overline{\mathrm{PGDP}}}_{\mathrm{i}}$ is the average per capita GDP of city $\:\mathrm{i}$during the study period (2014–2023). The matrix is row-standardized ($\:\mathrm{i.e.,}\sum\:_{\mathrm{j}}{\mathrm{w}}_{\mathrm{ij}}\mathrm{=1}$).

The Spatial Durbin Model (SDM), the most general form incorporating spatial lags of both the dependent and independent variables, is specified as follows:

4 $$\mathbf{Y}=\rho\mathbf{WY}+\mathbf{X}\boldsymbol{\beta}+\boldsymbol{\theta}+\epsilon$$

Here $\:\mathrm{Y}$ is the $\:\mathrm{n}\times1$vector of the dependent variable; $\:\mathrm{X}$ is the $\:\mathrm{n}\times 1$ matrix of explanatory variables; $\:\mathrm{WY}$ is the spatial lag of the dependent variable, with coefficient *ρ* measuring the strength of spatial dependence; $\:\mathrm{WX}\text{}$ is the spatial lag of the explanatory variables, with coefficient vector $\:\theta$ capturing the spatial spillover effects of the explanatory variables; *β* is the $\:K\times 1$ vector of coefficients for the local effects of the explanatory variables; and $\:\epsilon$ is the $\:\mathrm{n}\times1$ vector of random error terms.

3. Model selection and determination procedure

To ensure the scientific rigor and robustness of the model specification, a standard panel OLS model without spatial effects is first estimated, and a global Moran’s I test is conducted on its residuals. A significant test result indicates the presence of spatial autocorrelation, necessitating the use of spatial econometric models. After confirming the need to introduce spatial effects, the most general form, the Spatial Durbin Model (SDM), is estimated. Subsequently, Wald tests and Likelihood Ratio (LR) tests are performed to determine whether the SDM can be simplified to the Spatial Lag Model (SLM, i.e., ***θ*** = 0) or the Spatial Error Model (SEM, i.e., $\:\theta\mathrm{+}\rho\beta\mathrm{=0}$).If both null hypotheses are rejected, the SDM is considered the most appropriate model. Furthermore, the Hausman test is used to assess whether individual effects are correlated with the explanatory variables. A significant result supports the selection of a fixed effects model to control for time-invariant individual heterogeneity; otherwise, a random effects model is chosen.

## Results

### Global spatial agglomeration patterns

To systematically reveal the evolutionary trajectory of the spatial pattern of sports and recreation facilities in Jiangsu Province and its interaction with macro-policy cycles, four years were selected as analytical cross-sections within the 2014–2023 time window, based on temporal continuity and the representativeness of key policy nodes: 2014 (starting point of the study period), 2015 (baseline before the implementation of the National Fitness Strategy), 2018 (mid-point of the observation period), and 2023 (end of the observation period).

The global spatial agglomeration patterns for these years were analyzed using the Average Nearest Neighbor (ANN) index, with results presented in Table 3.

**Table 3:** Annual comparison of nearest neighbor analysis parameters

Year	Mean observed distance (m)	Mean expected distance (m)	Nearest neighbor ratio	z-score	*p*-value
2014	778.97	3147.08	0.248	−97.68	< 0.001
2015	412.63	1867.16	0.221	−175.24	< 0.001
2018	386.16	1799.28	0.215	−182.91	< 0.001
2023	373.39	1621.83	0.230	−200.92	< 0.001

The results indicate that the spatial distribution of sports and recreation facilities in Jiangsu Province exhibited a highly significant agglomerated pattern throughout the observation period (*p* < 0.001 for all years). Regarding temporal evolution, the agglomeration intensity displayed a dynamic characteristic of first intensifying and then slightly adjusting: the Nearest Neighbor Ratio (R-value) consistently decreased from 0.248 in 2014 to 0.215 in 2018, indicating that the spatial agglomeration effect of facility layout reached its peak by the mid-point of the observation period. In 2023, the R-value slightly rebounded to 0.230. This may suggest that, following the earlier phase of rapid concentration and driven by provincial-level coordinated development strategies, facility construction began to exhibit initial, guided diffusion outside the core hotspot areas. However, due to the strong inertia of historical accumulation and market forces, this diffusion remains limited and has not yet reversed the fundamental pattern of high-density in the south and low-density in the north, nor its highly agglomerated nature across the province.

### Evolution of local agglomeration morphology

**Fig. 4:**
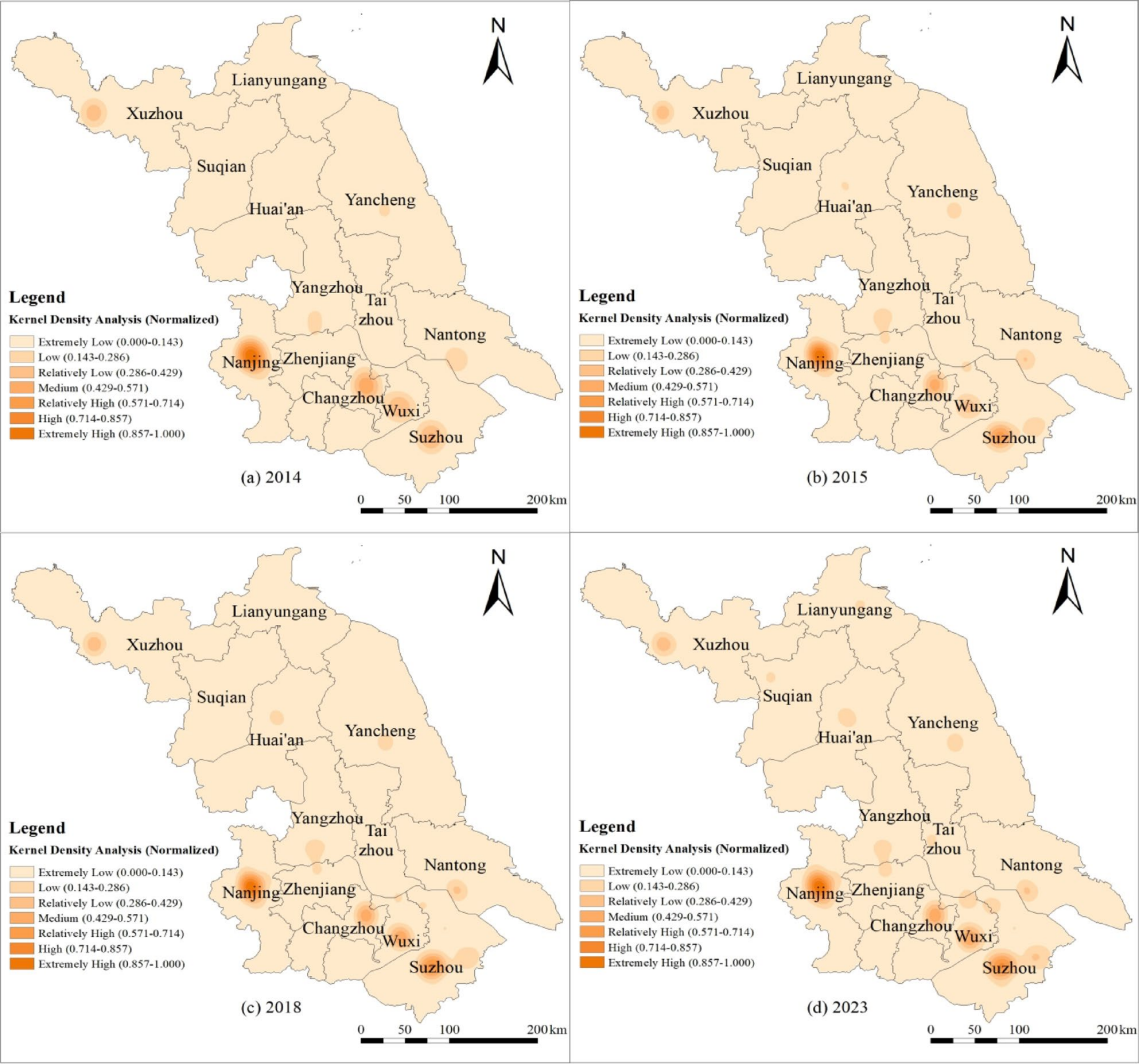
Kernel density estimation of sports and recreation facilities in jiangsu province (2014, 2015, 2018, 2023). Darker colors indicate higher density. Southern Jiangsu consistently served as the high-density core area, secondary hotspots gradually formed in the riverside areas of Central Jiangsu, while Northern Jiangsu maintained relatively low density overall with localized growth

While the Average Nearest Neighbor index reveals the macro-level temporal trends of sports and recreation facilities in Jiangsu Province, this global indicator cannot answer key questions such as where agglomeration occurs or how hotspot areas evolve. To further uncover the internal heterogeneity of facility distribution and the spatiotemporal evolution of local agglomeration patterns, Kernel Density Estimation (KDE) was employed for visualization analysis (Fig. 4).

The results reveal that high-density core areas remained persistently concentrated in Southern Jiangsu, exhibiting a significant “point-axis diffusion” characteristic. Hotspots are not uniformly distributed but are instead highly attached to high-end functional zones of major cities, such as Suzhou Industrial Park, Nanjing Hexi New Town, Wuxi Taihu New City, and the riverside new districts of Changzhou and Zhenjiang. Over the decade, the extent of these core areas continuously consolidated and experienced limited expansion, showing a trend of sprawl. This has reinforced the absolute status of the Southern Jiangsu urban agglomeration as the “peak area” for sports and recreation facilities in the province. Secondary hotspot areas gradually formed in the riverside belt of Central Jiangsu, reflecting the policy-driven diffusion along the Yangtze River corridor. Particularly after 2018, the density increased markedly in areas centered around the main urban districts of Nantong, Yangzhou, and Taizhou. These areas have formed a “transition zone” connecting the Southern Jiangsu core area with the vast Northern Jiangsu region. Although peripheral low-density areas cover most of Northern Jiangsu, clear signs of “growth pole emergence” appeared. Besides Xuzhou’s urban area, which consistently maintained relatively significant agglomeration as a regional center, the downtown areas of Yancheng, Huai’an, and Lianyungang, as well as central towns in some counties, began to show sporadic yet identifiable density growth points on the 2023 KDE map. This indicates that facility diffusion is not a uniform process but preferentially selects administrative centers or key towns as “policy anchors” for implantation.

In summary, the KDE analysis confirms, from a morphological and locational perspective, that the “limited diffusion” identified by the global analysis is essentially a highly selective spatial process. It manifests primarily as the marginal sprawl and ribbon development of high-density core areas in Southern Jiangsu, gradient transmission along the Yangtze River corridor to Central Jiangsu cities, and the pole-like emergence around regional central cities or county centers in Northern Jiangsu. This spatial pattern profoundly reflects the complex interplay between market forces and regional coordination policies in shaping facility layout.

### Macro-level distributional trends

**Fig. 5:**
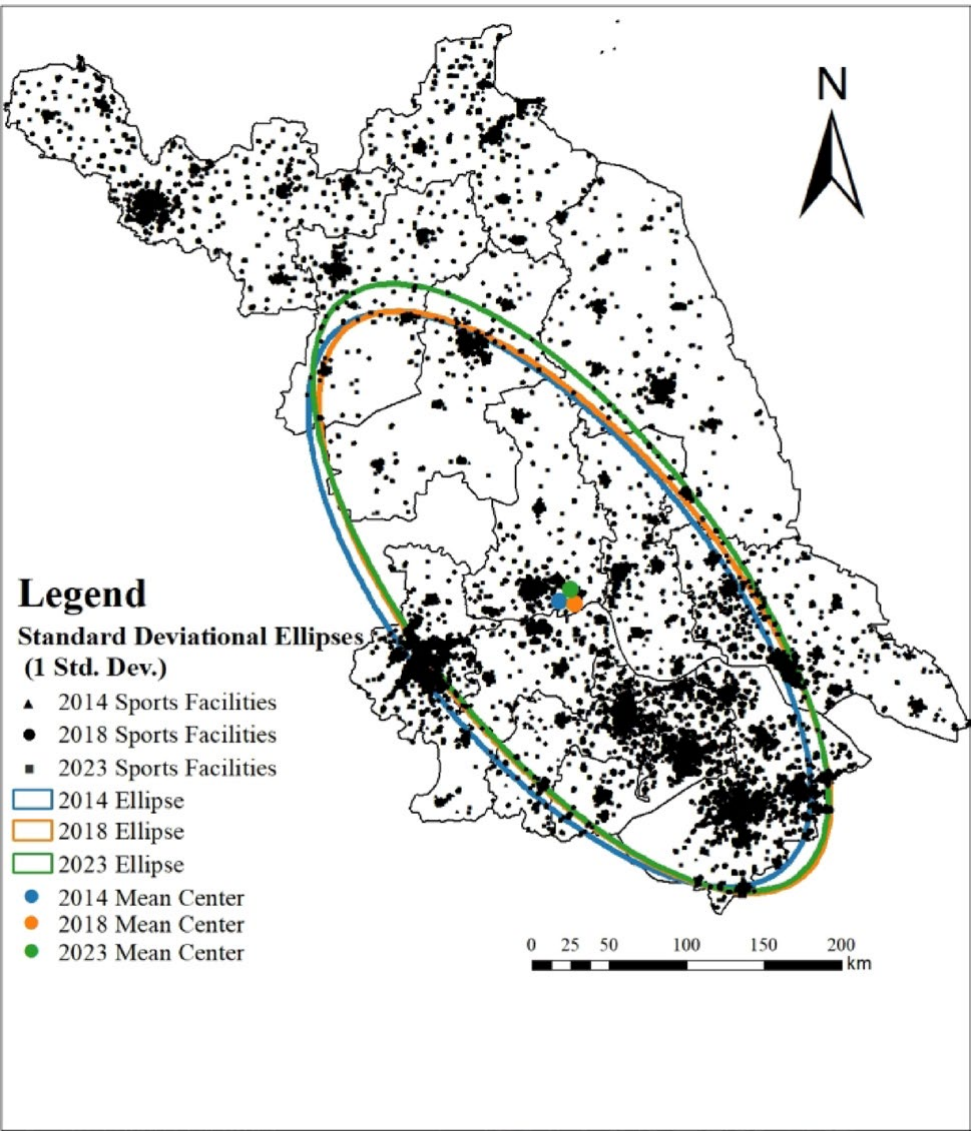
Standard Deviational Ellipses of Sports and Recreation Facilities in Jiangsu Province (2014, 2018, 2023). The ellipse coverage represents the main distribution areas of facilities, centroid migration reflects the movement trajectory of the overall distribution center, and changes in ellipse morphology indicate the direction of spatial expansion and the degree of spatial balance

Building on the local agglomeration details revealed by KDE, the Standard Deviational Ellipse (SDE) method was further employed, based on data for 2014, 2018, and 2023, to capture the overall direction, scope, and evolutionary process of the spatial distribution of sports and recreation facilities in Jiangsu Province from a macro-geometric perspective (Fig. 5; Table 4). The results show that the distribution pattern underwent a transformation from “linear agglomeration” to “balanced expansion” between 2014 and 2023.

**Table 4:** Annual comparison of standard deviational ellipse parameters

Year	Center coordinates(°)	Major axis(°)	Minor axis(°)	Ellipse area(°²)	Rotation angle(°)
2014	(119.574, 32.324)	1.857	0.829	4.837	133.81
2018	(119.662, 32.311)	1.896	0.812	4.839	133.64
2023	(119.637, 32.382)	0.833	1.945	5.091	135.05

All ellipse parameters are calculated based on the WGS84 geographic coordinate system. Center coordinates, major axis, and minor axis are measured in decimal degrees (°). Ellipse area is measured in square degrees (°²), representing a relative measure of spatial extent before geographic projection. These parameters collectively reflect the distribution center, orientation, and dispersion within the geographic coordinate space

Specifically: (1) Stable centroid and directional fine-tuning: The distribution centroid remained consistently near Taizhou, with the orientation angle (major axis direction) maintained between 133° and 135° (northwest-southeast direction), consistent with the Yangtze River’s orientation and the core economic corridor, confirming the core status of Southern Jiangsu. (2) Axis length reversal and pattern reconstruction: The most significant change occurred between the major and minor axes. From 2014 to 2018, the pattern exhibited a linear agglomeration form with the “major axis significantly longer than the minor axis.” By 2023, the minor axis length (1.945°) surpassed the major axis (0.833°), indicating that the dominant direction of expansion shifted from a single southeast-northwest corridor to a more balanced north-south expansion. (3) Continuous expansion of coverage range: The ellipse area steadily increased, intuitively reflecting an overall diffusion trend in the spatial coverage of facilities.

In summary, the SDE analysis reveals from a macro-geometric perspective that the spatial pattern of sports and recreation facilities in Jiangsu Province is transitioning from the highly linear agglomeration along the Yangtze River Economic Belt in the early stage towards a more extensive and directionally balanced networked distribution. This macro-level “from-line-to-area” reconstruction trend mutually corroborates the local processes of “core consolidation, corridor transmission, and growth pole emergence” observed in the KDE analysis. Together, they reveal the structural changes in facility allocation driven by the pursuit of spatial balance under the influence of regional coordinated development strategies.

### Testing the driving mechanisms of the spatial pattern

The preceding analysis has systematically revealed the spatial patterns and evolutionary characteristics of sports and recreation facilities in Jiangsu Province from three perspectives: global patterns, local morphology, and macro-level trends. However, this only answers the question of “what the patterns are.” To uncover the underlying driving factors and the interaction mechanisms between regions, this study further constructs spatial econometric models [42, 43].

#### Specification and testing of spatial econometric models

To select the appropriate model, a global Moran’s *I* test was first conducted on the residuals of the ordinary panel regression model. The results (Table 5) show that the Moran’s *I* indices for all years are significantly positive at the 1% level. It confirms the presence of significant spatial dependence in the distribution of sports and recreation facilities, necessitating the use of spatial econometric models.

**Table 5:** Global moran’s I test results (2014–2023)

Year	I	Z	*P*-value	Year	I	Z	*P*-value
2014	0.3689**	2.4631	0.0138	2019	0.2910**	2.1465	0.0318
2015	0.2904**	2.1298	0.0332	2020	0.3409**	2.4847	0.0130
2016	0.2557**	1.9785	0.0479	2021	0.2962**	2.0843	0.0371
2017	0.3598***	2.5857	0.0097	2022	0.2785**	2.0774	0.0378
2018	0.2924**	2.1682	0.0301	2023	0.2760**	2.1004	0.0357

***, **, and * denote statistical significance at the 1%, 5%, and 10% levels, respectively

After the need for spatial modeling was confirmed, the Spatial Lag Model (SLM), the Spatial Error Model (SEM), and the Spatial Durbin Model (SDM) were estimated. Subsequently, a series of statistical tests were conducted to determine the final model form, with results summarized in Table 6.

**Table 6:** Results of the spatial econometric model specification tests

Testing stage	Test	Statistic	*P*-value	Conclusion and implication
**Spatial dependence diagnostic**	Global Moran’s *I* (OLS residuals)	11.309***	0.000	Rejects OLS. Spatial models are required.
**Preliminary model screening**	Robust LM Test (Error Model)	48.568***	0.000	Initially supports SEM.
	Robust LM Test (Lag Model)	1.361	0.243	Initially rejects SLM.
**Final model determination**	Wald Test (SAR: θ=0?)	31.31***	0.000	Strongly rejects SLM. Supports SDM.
	Wald Test (SEM:*θ*+*ρβ*=0?)	30.76***	0.000	Strongly rejects SEM. Supports SDM.
	Likelihood Ratio Test (SAR)	28.12***	0.0001	Confirms SDM is the appropriate general form.
	Likelihood Ratio Test (SEM)	27.8***	0.0001	
**Effects type selection**	Hausman Test	90.32***	0.000	Rejects RE. Supports Fixed Effects.
**Joint tests for fixed effects**	LR Test (Individual Effects)	85.92***	0.000	Supports Spatial and Time Period Fixed Effects.
	LR Test (Time Effects)	112.52***	0.000	

***, **, and * denote statistical significance at the 1%, 5%, and 10% levels, respectively

As shown in Table 6, both the Wald test and the Likelihood Ratio (LR) test strongly reject the null hypothesis that “the SDM can be simplified to the SLM or SEM,” demonstrating that the Spatial Durbin Model (SDM), which includes spatial lags of the explanatory variables, is the optimal specification. Furthermore, the Hausman test supports the use of a fixed effects model. Consequently, the Spatial Durbin Model (SDM) with two-way fixed effects is adopted.

5 $$Y=\rho WY+X\beta+WX\theta+\mu+\lambda t+\epsilon$$

where ρ is the spatial autoregressive coefficient, W is the economic-geographic nested weight matrix, WY and WX are the spatial lags of the dependent and independent variables. μ and λt control for individual and time fixed effects, respectively.

#### Spatial durbin model results and effect decomposition

Since the coefficients (*β*,*θ*) of the SDM do not directly represent the true marginal effects of the explanatory variables, this study follows the method of LeSage and Pace [44] to decompose the total effects into direct effects (the impact of local factors on the local area) and indirect effects (i.e., spatial spillover effects, reflecting the impact of neighboring factors on the local area). The model estimation and effect decomposition results are presented in Table 7.

**Table 7:** Estimation results and effect decomposition of the spatial durbin model (SDM), 2014–2023

VARIABLES	Main	Wx	Spatial		Variance		Direct	Indirect	Total
*lnGDP*	0.437*	2.116***					0.475*	2.251***	2.726***
	(0.248)	(0.725)					(0.249)	(0.698)	(0.757)
*lnPopDen*	0.0210	−0.951*					−0.0313	−0.982**	−1.013***
	(0.358)	(0.503)					(0.301)	(0.480)	(0.306)
*lnFiscalExp*	−0.00500	−0.0914					−0.00523	−0.0990	−0.104
	(0.0210)	(0.0691)					(0.0229)	(0.0691)	(0.0781)
*RoadDen*	0.00773	−0.567***					0.00161	−0.599***	−0.598***
	(0.108)	(0.160)					(0.107)	(0.173)	(0.209)
*UrbanRate*	0.904*	0.122					0.809	0.304	1.113
	(0.532)	(1.009)					(0.622)	(1.221)	(1.394)
*lnIndEmp*	0.200**	−0.466**					0.199**	−0.480**	−0.281
	(0.0939)	(0.197)					(0.0942)	(0.229)	(0.274)
rho			0.0662						
			(0.114)						
sigma2_e					0.00728***				
					(0.000904)				
Observations	130	130	130		130		130	130	130
R-squared	0.593	0.593	0.593		0.593		0.593	0.593	0.593

***, **, and * denote statistical significance at the 1%, 5%, and 10% levels, respectively

The estimation results of the spatial econometric model provide a micro-level mechanistic explanation for understanding the previously described spatial patterns. The model reveals that the spatial dependence in sports and recreation facility allocation does not primarily stem from spatial imitation of the facilities themselves (*ρ* = 0.066, *p* = 0.563) but is more likely associated with the spatial interaction of socioeconomic factors (as indicated by the significance of multiple Wx terms). The effect decomposition further delineates a dynamic landscape characterized by “local agglomeration” and “regional interaction.”

First, economic growth exhibits a significant positive spatial spillover effect. The direct pull of economic development level (lnGDP) on local facility construction is limited (0.475, *p* < 0.1), but it demonstrates a highly significant positive spatial spillover (2.251, *p* < 0.01).This implies that areas adjacent to regions with higher economic development levels also tend to have higher levels of sports and recreation facility provision. This positive synergistic effect aligns with the phenomenon observed in the kernel density analysis of the continuous consolidation and peripheral diffusion of the core area in Southern Jiangsu. The agglomeration highlands formed by developed regions may benefit surrounding areas through channels such as consumption spillovers, policy demonstration, and knowledge diffusion.

Second, certain localized development factors exhibit different types of negative spatial spillovers while driving their own development. The analysis shows that related industrial agglomeration (*lnIndEmp*) has a significant positive impact on local facility supply, yet is accompanied by a significant negative spatial spillover (−0.480, *p* < 0.05). This result confirms the existence of “crowding-out effects,” where the prosperity of local industries coexists with constrained development in neighboring areas. For population density (*lnPopDen*), the local effect is insignificant, but the neighborhood effect is significantly negative (−0.982, *p* < 0.05). This aligns with the typical characteristics of a “siphon effect”: population agglomeration in surrounding areas may be associated with a relative outflow of local resources. The negative spillover of road density (*RoadDen*) (−0.599, *p* < 0.01) suggests a potential correlation between improved transportation accessibility in neighboring areas and relatively lagging local facility development. This pattern may be related to spatial substitution in residents’ consumption: when facilities in surrounding areas become more accessible, local service demand faces diversion.

Furthermore, both the direct and indirect effects of the urbanization rate (*UrbanRate*) are not statistically significant, indicating that its influence is largely confined within local administrative boundaries and has not yet formed significant spatial spillovers, exhibiting a typical “inward-oriented” characteristic. The various effects of local government fiscal capacity (*lnFiscalExp*) are also not significant in the model, suggesting that its moderating role in regional sports and recreation facility allocation has not yet been fully realized, and the guiding effect of fiscal investment on facility layout requires further activation.

In summary, the core driving factors examined in this study exhibit differentiated spatial spillover patterns. This configuration suggests that, at the current stage of development, the allocation of sports and recreation facilities in Jiangsu Province has not yet established an effective association with a “regional synergy” mechanism. It is precisely this spatial mode, dominated by siphon and crowding-out effects and lacking positive synergy, that provides a possible explanation for the high stability of the macro-spatial pattern. The “limited diffusion” observed earlier has not evolved into a fundamental pattern reconstruction, likely due to the inherently unbalanced and inward-agglomerating nature of the core forces driving development. This offers a valuable analytical perspective for understanding the long-term maintenance of the “high density in the south, low density in the north” gradient disparity.

## Conclusions and discussion

### Conclusions

Driven by the national strategies for coordinated regional development and National Fitness, the spatial allocation of public service facilities has become an important dimension for measuring the quality of regional development. This study, employing exploratory spatial data analysis and spatial econometric models, systematically investigated the evolutionary patterns and driving mechanisms of sports and recreation facility allocation in Jiangsu Province from 2014 to 2023. The main conclusions are as follows:

1. The distribution of sports and recreation facilities exhibits a stable “core-periphery” structure, yet shows signs of limited diffusion with differentiated pathways. The core area in Southern Jiangsu continuously consolidated and exhibited a sprawling trend. Central Jiangsu, leveraging the riverside corridor connected to the core area, formed relatively stable secondary hotspots. Northern Jiangsu remains largely in a low-level equilibrium state, but some prefecture-level cities have developed relatively agglomerated growth poles. The Standard Deviational Ellipse further reveals that the spatial pattern is transitioning from the early-stage linear agglomeration along the Yangtze River Economic Belt towards a broader, more directionally balanced “from-line-to-area” reconstruction.
2. The spatial externalities of different development factors exhibit significant heterogeneity. Economic growth (*lnGDP*) manifests as a positive spatial spillover, where areas adjacent to regions with higher economic development levels tend to have higher sports facility provision. Conversely, population density (*lnPopDen*), industrial agglomeration (*lnIndEmp*), and road density (*RoadDen*) exhibit negative spatial spillovers, characterized by “siphon effects,” “crowding-out effects,” and “consumption substitution,” respectively. This indicates that the spatial effects of population and industrial factors tend towards inhibition rather than diffusion.
3. The stability of the macro pattern is closely related to the dominant logic of regional interaction. When “siphon effects” and “crowding-out effects” become the primary modes of regional interaction, peripheral areas may persistently remain in a marginal position. The long-term maintenance of the spatial pattern is the outcome of the interplay among multiple forces from the demand side, supply side, and environmental support side.

### Discussion

The methodological framework of “pattern identification–mechanism testing–effect decomposition” followed in this study provides a progressive analytical pathway for spatial research on public service facilities. This progression, from macro-pattern description to micro-mechanism testing, contributes to a more systematic understanding of the formation logic of spatial phenomena.

The theoretical framework, constructed from the three dimensions of demand side, supply side, and environmental support side, corroborates the duality of spatial spillover effects. More precisely, when analyzing the spatial externalities of development factors, it is necessary to distinguish between two fundamentally different spillover mechanisms: “synergistic spillovers” and “negative spillovers”, which include siphon effects and crowding-out effects. This finding expands the understanding of the complexity of regional interaction mechanisms and offers new insights for regional sports facility planning: strengthening planning coordination within metropolitan areas, promoting infrastructure connectivity, and facilitating the co-construction and sharing of public services can help amplify the radiation effects of core cities [45–47]. However, in advancing regional coordinated development strategies, attention must be paid to potential development constraints in peripheral areas resulting from factor outflows. These could be mitigated through provincial-level fiscal transfers and strategic allocation of major facilities to offset negative spillover effects [48, 49].

Due to data availability, this study primarily constructs the explanatory variable system using objective statistical indicators at the prefecture-city level to ensure the validity of spatial econometric analysis. This, to some extent, limits the exploration of subjective dimensions such as residents’ satisfaction with facility usage, perceived health benefits, and accessibility experiences from a micro-individual perspective. Future research could integrate multi-source spatiotemporal big data, such as mobile phone signaling data, internet map heat indices, and social media check-ins, to further investigate the actual efficiency and spatial equity of facility usage. It could also incorporate resident questionnaire surveys to develop an indicator system combining subjective and objective measures, providing a more comprehensive picture of the spatial benefits of sports and recreation facilities. Furthermore, as the empirical analysis based on Jiangsu Province, future research could be extended to other regions in the Yangtze River Delta Region or a national scale. Through multi-case comparisons, it could reveal commonalities and differences in the spatial allocation patterns of sports and recreation facilities across different development stages and geographical environments, further exploring the mechanisms through which the spatial benefits of sports and recreation facilities influence regional sustainable development.

## Data Availability

The data that support the findings of this study are available from the following sources but restrictions apply to their availability: Point of Interest (POI) data for recreational sports facilities were derived from the Amap (AutoNavi) API under license and are not publicly available. Interested researchers may apply for access through the official Amap Open Platform.Socioeconomic statistical data were obtained from publicly available yearbooks and statistical bulletins published by municipal and provincial bureaus of statistics.Processed data and analysis code supporting the conclusions are available from the corresponding author on reasonable request.
